# Immediate extubation versus standard postoperative ventilation: Our experience in on pump open heart surgery

**DOI:** 10.4103/0019-5049.72641

**Published:** 2010

**Authors:** Srikanta Gangopadhyay, Amita Acharjee, Sushil Kumar Nayak, Satrajit Dawn, Gautam Piplai, Krishna Gupta

**Affiliations:** Asansol Subdivisional Hospital, Asansol, West Bengal, India; 1 Department of Anaesthesiology, Calcutta National Medical College, Kolkata - 14, India; 2 Department of Anaesthesiology, KPC Medical College, Kolkata, India

**Keywords:** Haemodynamic parameters, immediate extubation, off pump, on pump, postoperative complication, postoperative ventilation

## Abstract

Elective postoperative ventilation in patients undergoing “on pump” open heart surgery has been a standard practice. Ultra fast-track extubation in the operating room is now an accepted technique for “off pump” coronary artery bypass grafting. We tried to incorporate these experiences in on pump open heart surgery and compare the haemodynamic and respiratory parameters in the immediate postoperative period, in patients on standard postoperative ventilation for 8-12 hours. After ethical committee’s approval and informed consent were obtained, 72 patients, between 28 and 45 years of age, undergoing on pump open heart surgery, were selected for our study. We followed same standard anaesthetic, cardiopulmonary bypass (CPB) and cardioplegic protocol. Thirty-six patients (Group E) were randomly allocated for immediate extubation following operation, after fulfillment of standard extubation criteria. Those who failed to meet these criteria were not extubated and were excluded from the study. The remaining 36 patients (Group V) were electively ventilated and extubated after 8-12 hours. Standard monitoring for on pump open heart surgery, including bispectral index was done. The demographic data, surgical procedures, preoperative parameters, aortic cross clamp and cardiopulmonary bypass times were comparable in both the groups. Extubation was possible in more than 88% of cases (n=32 out of 36 cases) in Group E and none required reintubation for respiratory insufficiency. Respiratory, haemodynamic parameters and postoperative complications were comparable in both the groups in the postoperative period. Therefore, we can safely conclude that immediate extubation in the operating room after on pump open heart surgery is an alternative acceptable method to avoid postoperative ventilation and its related complications in selected patients.

## INTRODUCTION

Post operative ventilation of patients undergoing “on pump” cardiac surgery has been the standard practice for the last three decades because of a relatively high risk of respiratory insufficiency and low cardiac output state due to universal use of high dose opioid anaesthetic technique.[1] However, with the recent advancement in anaesthesia, surgery, myocardial protection, haemodynamic monitoring and postoperative analgesia, several authors have promoted operating room (OR) extubation after on pump cardiac surgery[2] and observed reduced resource utilisation, mortality rate and postoperative complications.[2–5] High-quality postoperative analgesia and quick rehabilitation was found with immediate extubation of the patients in the OR, using thoracic epidural analgesia (TEA) along with general anaesthesia.[2–5] We therefore conducted a prospective randomised study in which we compared haemodynamic, respiratory, pain control parameters and complications of patients, who had been extubated in the OR, with another group of patients who had been electively ventilated in the postoperative period after on pump cardiac surgery with TEA in both the groups.

## METHODS

After obtaining institutional ethical committee’s approval, we conducted this prospective, randomised study. Written informed consent was obtained from all patients for epidural catheter insertion and immediate extubation in the OR as well as elective ventilation in the postoperative period.

From June 2006 to July 2008, a total of 72 patients, aged between 28 and 45 years, undergoing on pump open heart surgery by one surgical team, were included in our study. Patients having a contraindication to the insertion of an epidural catheter[6] and who refused to participate were excluded from our study.

Preoperative anticoagulation with clopidogrel/warfarin was replaced with low molecular weight heparin (LMWH) 10 days before surgery and the last dose of LMWH was administered 12 hours before insertion of epidural catheter. (INR) >1.2 and activated partial thromboplastin time (APTT) were brought to normal values before the insertion of epidural catheter.

Anaesthesia was performed in the same fashion in all patients. Monitoring included electro cardio gram (ECG), pulse oximetry (SpO_2_), end tidal carbondioxide (EtCO_2_), invasive blood pressure using a radial artery catheter, central venous pressure using internal jugular venous access, arterial blood gas (ABG), core temperature (oesophageal and rectal), urine output, BIS (Aspect 2000 Monitoring System) and train of four (TOF watch, Organon, Dublin, Ireland). In all the patients, an epidural catheter was inserted at T_2_-T_3_ or T_3_-T_4_ interspace under local anaesthesia, at least 12 hours preoperatively. The epidural catheter was discontinued 48-72 hours after surgery.

All the patients were premedicated with tablet diazepam (0.2 mg/kg), 90 minutes before arrival in the OR. Before induction of anaesthesia, all monitors were attached and the epidural catheter was checked for proper placement in the epidural space. Anaesthesia was induced with fentanyl 5 μg/kg i.v., followed by administration of propofol 1-2 mg/kg. Endotracheal intubation was facilitated with i.v. administration of vecuronium 0.1 mg/kg. Intermittent positive pressure ventilation was started and adjusted to a rate to achieve EtCO_2_ in the range of 30-35 mm Hg. Anaesthesia was maintained with sevoflurane to achieve a BIS between 40 and 50. TOF was measured every 6 minutes; when TOF count was 1 or more, vecuronium (0.02 mg/kg) was administered. Intraoperative analgesia was provided with a bolus of 4-8 ml of 0.25% bupivacaine and 3 μg/ml of fentanyl citrate through epidural catheter, 15 minutes before skin incision, and thereafter, epidural infusion of bupivacaine (0.25%) at the rate of 10 ml/hour and fentanyl at the rate of 40 μg/hour using infusion pump. In the intraoperative period, if BIS was maintained between 40 and 50 and there was inadequate analgesia [increase in heart rate (HR) and mean arterial pressure (MAP)>20% of baseline value], the epidural infusion rate was increased. Conversely, when hypotension (MAP<60 mm Hg) was detected, the infusion rate was decreased in conjunction with fluid replacement. A circulating water mattress, air warmer, routine use of warmed fluid and increased OR temperature ≥22°C were used to maintain the core temperature within 35-37°C. An open cardiopulmonary bypass (CPB) with arterial filter was used. Heparinisation (300 IU/kg) of the patient was adjusted to maintain activated clotting time>400 seconds and antagonised using protamine 1.3 mg for 100 IU of heparin after completion. Blood flow was maintained between 2.4 L and 2.8 L/min/body surface area (BSA). Perfusion pressure was kept within the range of 60-70 mm Hg. The CPB temperature was maintained between 33 and 34°C and full rewarming to 37°C was achieved before weaning CPB. Intermittent whole blood cardioplegia from the arterial line of the CPB unit, supplemented with K^+^, Mg^+2^ and adenosine, was given in an antegrade or a retrograde manner. During the ischaemic period, bradycardia (HR < 40 beats/minute) was treated with increments of atropine 0.3 mg i.v. Hypotension (MAP<60 mm Hg) was treated with increments of phenylephrine 50 μg i.v.

At the end of the surgery, the patients were randomly allocated into two groups with an equal number of patients: Group V (*n*=36) and Group E (*n*=36). Patients of Group V were electively ventilated in the postoperative period for 8-12 hours using propofol for sedation and endotracheal tube tolerance. In Group E, at the end of surgery, neuromuscular block was reversed with neostigmine 0.05 mg/kg and glycopyrrolate 0.01 mg/kg and patients were extubated in the OR. The standard extubation criteria were as follows: (i) cooperative and alert patient; (ii) smooth spontaneous ventilation; (iii) sustained head lift and TOF>0.8 at the adductor pollicis; (iv) SpO_2_>96% on FIO_2_of 1, EtCO_2_<45 mm Hg; (v) stable haemodynamics; (vi) core temperature ≥35°C and (vii) no evidence of early surgical complications.

Postoperative epidural infusion rate was increased by 1ml/hour every 2 hours, along with a bolus of 3-5 ml of solution (bupivacaine 0.125% and fentanyl 3 μg/ml) was administered in both the groups in case of inadequate analgesia (determined by increase in MAP and HR 20% above normal and on patient demand).

Postoperatively, HR, blood pressure and respiratory rate were recorded every 2 hours after surgery. Complications such as reintubation, bleeding, haemodynamic problems (bradycardia, hypotension, arrhythmia, need for cardiac pacing) and respiratory dysfunction (PaO_2_, PaCO_2_) were also noted. Complications such as nausea, pruritus and episodes of paraesthesia were also recorded.

Group size was calculated to achieve a power of more than 90%. We calculated a group size of 20 patients to show at least a 20% difference of MAP and HR, 4 hours after surgery, with a power of 0.8. Intergroup comparison was done by applying *F* test and unpaired two sample “*t*” test as appropriate. A *P* value of <0.05 was considered significant.

## RESULTS AND ANALYSIS

There were no significant differences in age, sex, weight, ejection fraction, preoperative medical conditions, surgical procedures and CPB time between the two groups [Tables 1 and 2]. Room and body temperature at the beginning and at the end of surgery were comparable between the two groups. The insertion of thoracic epidural catheter was successful in all the patients. The catheter was taken out at 56 hours (SD 5) without any group related difference. Out of the 36 patients in Group E, 32 patients (89%) satisfied the protocol of extubation and were successfully extubated in OR, and no patient required reintubation and ventilation within 24 hours. Out of the rest of the four patients, two with low core temperature, one with impaired consciousness (due to transient cerebral ischaemia) and the other one with an HR>120/minute were not extubated in the OR. These patients were ventilated postoperatively in the intensive care unit and extubated within 4 hours after satisfying the protocol for extubation. The mean (SD) values of PaO_2_ (on FIO_2_ of 1.0) immediately after extubation in Group E was 247 (63) mm Hg and in Group V it was 242 (61) mm Hg. The mean (SD) values of PaCO_2_ in Group E immediately after extubation and in Group V after the end of surgery were 39.75 (5.25) mm Hg and 38.25 (3.75) mm Hg, respectively. These values were not statistically different. Moreover, PaO_2_ and PaCO_2_ values estimated at different intervals postoperatively were also comparable in the two groups [Table 3].

**Table 1:** Comparison of diseases and surgical procedures

Diseases	Group V (*n*=36)	Group E (*n*=36)	*P* value
ASD	8	10	
VSD	10	11	>0.05
Mitral valve replacement	8	6	
Aortic valve replacement	6	4	
Others	4	3	

ASD - ???, VSD - ???

**Table 2:** Comparison of demographic data, preoperative medical conditions, ejection fraction, pulmonary arterial pressures, aortic cross clamp and cardiopulmonary bypass times

Profiles	Group V	Group E	*P* value
Age in years [mean (range)]	23.1 (28-35)	26.3 (28-38)	
Males/females	17/19	17/15	
Diabetes mellitus	0	0	
Hypertension	0	0	
NYHA class III	4	5	>0.05
EF	51%	53%	
PAP (mm Hg) mean (range)	32 (10-76)	36 (12-70)	
Aortic cross clamp time (minutes) (mean)	63	67	
CPB time (minutes) (Mean)	76	81	

EF - Ejection fraction, PAP - Pulmonary arterial pressures, CPB - Cardiopulmonary bypass

**Table 3:** Comparison of ABG values between the two groups (mean±SD)

Time points	ABG	Group V	Group E	*P* value
Pre operative	PaO_2_	88.6±6.8	89.2±12.1	>0.05
	PaCO_2_	42.5±2.0	41.9±2.9	>0.05
10 minutes after extubation	PaO_2_	196.6±12.4	199.5±6.2	>0.05
	PaCO_2_	45.2±3.1	44.0±3.2	>0.05
30 minutes after extubation	PaO_2_	126.7±10.6	130.8±10.9	>0.05
	PaCO_2_	35.4±6.6	36.4±3.8	>0.05
60 minutes after extubation	PaO_2_	116.6±9.2	115.4±6.9	>0.05
	PaCO_2_	32.1±6.2	33.4±4.1	>0.05
120 minutes after extubation	PaO_2_	108.4±10.6	110.6±12.3	>0.05
	PaCO_2_	30.2±2.1	29.5±3.2	>0.05
6 hours after extubation	PaO_2_	126.4±12.9	122.7±6.7	>0.05
	PaCO_2_	32.4±1.2	33.0±2.1	>0.05

ABG - Arterial blood gas

There was no intraoperative bradycardia in any group. Increments of phenylephrine were used during ischaemia in 15 patients in Group E and 12 patients in Group V, which was found to be statistically insignificant. Mean blood pressure and HRs were stable and did not differ statistically between the two groups in the postoperative period [Table 4]. The rates of epidural infusion were also comparable.

**Table 4:** Comparison of HR and MAPs between the groups (mean±SD)

Time points	Group V			Group E		
	HR	MAP	*P* value	HR	MAP	*P* value
Preoperative values	78.3±8.6	96.2 ± 7.1	>0.05	76.2±7.1	94.6±12.6	>0.05
10 minutes after extubation	110.3±6.8	102.6±12.4	>0.05	112.4±12.1	106.6±10.2	>0.05
30 minutes after extubation	98.4±2.6	98.4±6.9	>0.05	96.0±7.5	96.4±7.5	>0.05
60 minutes after extubation	88.4±6.8	96.8±6.0	>0.05	85.7±7.6	96.0 ±3.1	>0.05
120 minutes after extubation	82.6±12.7	92.1±10.2	>0.05	84.7±8.7	90.3±6.1	>0.05
6 hours after extubation	78.4±6.1	88.2±10.2	>0.05	76.4±5.3	84.4±6.3	>0.05

HR - Heart rate, MAP - Mean arterial pressure

The frequency of postoperative complications was comparable in the two groups [Table 5]. No patient suffered from respiratory insufficiency after extubation or postoperative myocardial infarction. A transient atrial fibrillation occurred in 14 patients in Group E and 12 patients in Group V with no statistical difference and was managed in all patients with synchronous defibrillation. Three patients in each group required reintubation 24 hours after extubation. Three patients in Group E and two in Group V were reintubated due to unstable haemodynamics and required reoperation due to mediastinal bleeding. One patient in Group V required reintubation, three days after operation, due to chest infection and respiratory insufficiency. Seven patients in Group V and six in Group E required postoperative ionotropic support. There was no perioperative death in either group.

**Table 5:** Comparison of postoperative complications between the two groups

Complications	Group V	Group E
Wound infection	2*	3*
Blood transfusion	24*	22*
Respiratory failure	0	0
Postoperative atrial fibrillation	12*	14*
Postoperative myocardial infarction	0	0
Postoperative ionotropic support	7*	6*

* None are significantly different at 95% level of significance

Side effects related to epidural analgesia were similar in both the groups. Twenty percent patients in Group E and 23% patients in Group V complained of paraesthesia in dermatomes T1 and C8. In these patients, paraesthesia subsided on lowering the epidural infusion rate. Two patients in Group E and one in Group V complained of pruritus. The study solution was changed to plain bupivacaine. All the patients were awake during the study period. No patient in either group showed neurological signs and symptoms of epidural haematoma. There was no difference between the two groups in the incidence of nausea and vomiting. No patient required supplementary analgesia.

## DISCUSSION

To date, there has been no study comparing the patients of fast-track extubation with patients of elective postoperative ventilation after on pump cardiac surgery. In this study, we applied TEA in both the groups of patients and found no difference in respiratory and haemodynamic parameters as well as complications. However, patients extubated in the OR were awake immediately after surgery and had less chest infection compared to ventilated group. TEA provided high-quality perioperative analgesia in all patients. Our study corroborates with the findings of other studies.[2–5]

Edgerton and others[7] observed that patients immediately extubated after off pump coronary artery bypass grafting had a reduced incidence of atrial fibrillation, shorter length of stay and also reduced mortality. However, few authors argued against the early extubation, immediately after cardiac surgery. Their views are that immediate extubation activates the sympathetic nervous system, thereby causing haemodynamic instability and myocardial ischaemia.[8] Borracci and others[9] suggested that immediate extubation after on pump and off pump cardiac surgery should be avoided in patients with heart failure, left ventricular dysfunction, cross-clamping time, pacemaker usage, haemodynamic compromise and difficult cardiopulmonary bypass weaning. The chances of requiring reintubation are increased if the patients are haemodynamically unstable, cold, hypovolaemic or had considerable opioid medication.[2] In the present study, patients with core body temperature <35°C, haemodynamically unstable, requiring pacemaker during intraoperative period or prolonged CPB weaning time were not attempted for immediate extubation after surgery and were excluded.

Prospective studies by Royse and others[2] and Hemmerling and others[5] using TEA or opioid based analgesia in patients undergoing on pump or off-pump cardiac surgery showed that immediate extubation in OR was possible with both the techniques of pain control when normothermia was maintained. However, Hemmerling and others[5] had the opinion that as there are more adverse effects such as confusion, sedation, intestinal ileus or respiratory depression with morphine based patient controlled analgesia, TEA should be the preferred modality of pain control due to better postoperative analgesia with reduced incidences of pulmonary complications, thereby improving postoperative lung function[10] and perioperative arrhythmias.[11] Moreover, TEA can produce cardiac sympatholysis which increases coronary blood flow, decreases myocardial oxygen consumption[12] and reduces the incidences of postoperative myocardial infarction linked to tight stenosis and ratio of delivery to utilization of Oxygen (DO_2_/VO_2_) imbalance.[13] TEA also provides good protection from stress response and ensures haemodynamic instability.[14]

With the use of TEA as perioperative analgesia and by maintaining body temperature to >36°C in the OR, our results demonstrated no statistically significant increase in the rate of reintubation, haemodynamic instability, arrhythmias, myocardial infarction or respiratory complications compared to patients ventilated for 8-12 hours postoperatively after cardiac surgery.

There is controversy regarding the use of TEA in full anticoagulation during CPB due to risk of epidural haematoma. It is more likely if the epidural catheter is inserted or removed while the patient is anticoagulated.[15] We followed the guidelines of neuraxial anaesthesia in patients receiving antithrombotic therapy adopted from Horlocker and others[6] In our study, we inserted the epidural catheter at least 12 hours before operation and removed the catheter after the procedure when coagulation had returned to normal (INR>1.5). This practice was also followed by other investigators.[15] We did not observe any neurological complications due to TEA. Several other studies using TEA for cardiac surgery support this observation.[2,3]

Epidural analgesia is therefore an important adjunct to immediate extubation after open heart surgery because analgesia is optimised, patients remain awake and are not depressed by use of lots of narcotics, and early mobilisation with restoration of normal physiological function is possible.

Our limitation was that we could not use transoesophageal echocardiography during CPB for direct visualisation of left ventricular filling, detection of abnormal ventricular function, haemodynamic instability and new regional wall motion abnormalities. Moreover, as we studied two groups of patients, one on postoperative ventilation and thereby sedated, and another group conscious and awake after surgery, we could not use visual analogue scale (VAS) for assessment and comparison of pain.

Compared to conventional techniques, the potential benefits of early extubation in OR after cardiac surgery were reduced airway and lung trauma, improved cardiac output and renal perfusion with spontaneous respiration, reduced stress and discomfort of endotracheal tube suctioning and weaning from ventilation. There was no need for sedative drugs, ventilator disposables could be avoided and patients were transferred to lower dependency ward early from post anaesthesia care unit (PACU). Moreover, fewer nursing staffs were required to manage each patient. By this, cost saving also was therefore possible.

## CONCLUSION

Thus, immediate extubation after on pump cardiac surgery may be safely achieved with optimisation of perioperative analgesia with TEA and by maintaining normothermia, with several advantages.
